# Agreement between Force Platform and Smartphone Application-Derived Measures of Vertical Jump Height in Youth Grassroots Soccer Players

**DOI:** 10.3390/sports11060117

**Published:** 2023-06-13

**Authors:** Jason Tallis, Rhys O. Morris, Michael J. Duncan, Emma L. J. Eyre, Lucas Guimaraes-Ferreira

**Affiliations:** 1 Centre for Physical Activity, Sport and Exercise Science, Coventry University, Coventry CV1 5FB, UK; 2 School of Life Sciences, Coventry University, Coventry CV1 5FB, UK

**Keywords:** vertical jumps, testing, mobile device, soccer, physical performance

## Abstract

Given the importance of vertical jump assessments as a performance benchmarking tool, the assessment of neuromuscular function and indicator of health status, accurate assessment is essential. This study compared countermovement jump (CMJ) height assessed using MyJump2 (JH_MJ_) to force-platform-derived jump height calculated from time in the air (JH_TIA_) and take-off velocity (JH_TOV_) in youth grassroots soccer players. Thirty participants (Age: 8.7 ± 0.42 yrs; 9 females) completed bilateral CMJs on force platforms whilst jump height was simultaneously evaluated using MyJump2. Intraclass correlation coefficients (ICC), Standard error of measurement (SEM), coefficient of variance (CV) and Bland–Altman analysis were used to compare performance of MyJump2 to force-platform-derived measures of CMJ height. The median jump height was 15.5 cm. Despite a high level of agreement between JH_TIA_ and JH_TOV_ (ICC = 0.955), CV (6.6%), mean bias (1.33 ± 1.62 cm) and 95% limits of agreement (LoA −1.85–4.51 cm) were greater than in other comparisons. JH_MJ_ performed marginally better than JH_TIA_ when compared to JH_TOV_ (ICC = 0.971; 95% CI’s = 0.956–0.981; SEM = 0.3 cm; CV = 5.7%; mean bias = 0.36 ± 1.61 cm; LoA = −3.52–2.80 cm). Irrespective of method, jump height did not differ between males and females (*p* > 0.381; *r* < 0.093), and the comparison between assessment tools was not affected by sex. Given low jump heights achieved in youth, JH_TIA_ and JH_MJ_ should be used with caution. JH_TOV_ should be used to guarantee accuracy in the calculation of jump height.

## 1. Introduction

Vertical jump assessments are used regularly in sports and exercise settings due to their ease of implementation and broad and population-spanning application. Vertical jump performance provides an indication of lower limb power [1] and is used as a benchmarking tool for injury screening and rehabilitation [2,3], to evaluate the neuromuscular function and fatigue [4], and as a health marker [5]. Vertical jump performance is of particular significance for youth, given the importance of evaluating lower limb power and neuromuscular function as markers of movement and physiological development [6,7,8], highlighting a need for accurate assessment.

Several assessment tools can be used to determine vertical jump performance. Methods that derive jump height from time in the air (TIA), such as contact platforms [9], high-speed cameras [10], photoelectric cell systems [11] and accelerometers [12] are commonly used due to their portability and ease of data acquisition. Despite evidence demonstrating good test–retest reliability of jump height determined from such methods in youth [13,14,15], the comparison to the gold standard assessment of jump height, determined from force-platform-derived take-off velocity (TOV) [16], has received little attention, particularly from youth. Comparisons between assessment tools and calculation methods are important to evaluate the accuracy and suitability of jump height methods. Given the low jump heights achieved, such approaches are particularly important in youth, as seemingly small magnitudes in absolute error are likely to have profound effects on the sensitivity of the assessment tool to differentiate between performers and detect change, given the low jump heights in youth.

Although force platforms are becoming more portable and affordable, they are still not widely accessible and, in many cases, result in timely data acquisition. As such, there is a need for low-cost, time-efficient, user-friendly alternatives to accurately measure vertical jump height. One such assessment tool that has received recent attention is the smartphone application MyJump, which uses the device’s in-built high-speed camera to determine jump height from TIA. Previous evidence, which for the most part is specific to adult populations, demonstrates acceptable validity when compared to other TIA-derived assessment methods (i.e., force plates, contact mats, photoelectric cell systems) [17,18,19,20,21,22,23]. Only two studies have evaluated the performance of the MyJump app in youth. In a population of school-aged children aged 12.3 ± 0.8 yrs, Bogataj et al. [19] demonstrated that both squat jump and countermovement jump (CMJ) height were valid when compared to vertical jump performance measured using the OptoJump photoelectric cell system. In agreement with the adult literature, Rogers et al. [24] demonstrated extremely high agreement between MyJump and force-plate-derived CMJ height derived from TIA in a population of junior athletes (aged 15.0 ± 1.4 yrs). Whilst collectively previous work offers insight with respect to the performance of MyJump compared to other assessment tools, this work does not consider performance of the smartphone application to gold standard assessments of vertical jump height derived from TOV which can be achieved from mathematical integration of force platform data. Irrespective of the device used, it has long been established that jump height is influenced by calculation method, where determining take-off velocity, and subsequently jump height, using the impulse momentum method is recommended given the increased accuracy compared to TIA-derived jump height [25]. Both TIA- and TOV- derived vertical jump height are calculated based on the kinematic equations for free fall where known variables of acceleration due to gravity, time (in the case of jump height derived from TIA), and velocity (in the case of jump height derived from TOV) are used to calculate vertical displacement of the body’s centre of mass using equations of motion. The calculation of jump height from TIA assumes that the position of the body at take-off (i.e., extension at hip, knee and ankle), is identical to that at landing, and any manipulation in landing kinematics will result in an increased TIA and an overestimation of jump height [16]. Given TOV is determined during the contact phase of a vertical jump, the performance error that may occur from TIA methods is not prevalent.

To date, only two studies using adult populations have examined the validity of the MyJump smartphone application compared to the gold-standard assessment of vertical jump height using force-plate-derived TOV and have reported contrasting results, with direct comparisons revealing ICCs ranging from −0.026 (poor) to 1.00 (excellent) [18,26]. Given that vertical jump movement patterns are far less developed and repeatable in youth [27], TIA-derived jump height may be more erroneous in youth populations and as such, less appropriate. Given the importance of vertical jump assessments for youth and the dearth of literature examining the validity of commonly used TIA-derived vertical jump height measures compared to gold standard force-plate-derived TOV measures of jump height, the present study aimed to uniquely examine the agreement between CMJ height measured from MyJump2 smartphone application and that derived from force platforms (determined by both TIA and TOV) in youth grassroots soccer players.

## 2. Materials and Methods

### 2.1. Participants

Following ethics approval from the host institute, written parental consent and participant assent, 30 youth grassroots soccer players (*n* = 21 male; *n* = 9 female; Mean ± SD; Age: 8.7 ± 1.3 yrs; Height 139.6 ± 6.6 cm; Body mass 33.0 ± 2.8 kg) completed the study. Participants were free from contradictions to exercise, were competing and/or training at least twice per week and were familiar with assessments of CMJ as part of previous work with our research group. Participants were excluded if they had experienced a lower limb injury in the last 6 months, were rehabilitating from a previous lower limb injury, or had either an acute or chronic medical condition that effected safe completion of the test. Participants attended the human performance laboratories at the host institute on a single occasion to complete the assessments outlined below.

### 2.2. Experimental Procedures

Each participant completed three CMJ assessments whilst standing on two uni-axial force plates (PASCO, Pasport PS-2142, Roseville, CA, USA) sampling at 1000 Hz. Participants were asked to stand as still as possible for approximately 5 s to obtain a stable force trace before executing the jump. Participants were asked to jump as high as possible with arms akimbo (fixed on the hips) [5], and participants wore their own athletic training shoes whilst completing the assessments. Before collecting data, participants completed 3–5 familiarisation attempts, following a demonstration, to ensure the correct technique. Participants were informed that their legs should remain straight during flight, making contact with the ground with the tips of their feet and knees extended [5]. This was checked visually during the familiarisation assessments and experimental trials.

Simultaneously, CMJ performance was captured and analysed using the MyJump2 smartphone application (© Carlos Balsalobre-Fernández, http://www.myjumplabpro.com/ (accessed on 1 June 2023)), that uses video analysis to determine jump height from TIA. The inbuilt high-speed video of an iPhone 13 (Apple, Cupertino, CA, USA) sampling at 240 Hz recorded the CMJ. As per previous work, video capture was performed with the experimenter lying prone, ~1.5 m away from the force platforms and zooming in on the feet [17]. Two independent observers with video analysis experience and a background in biomechanics research both independently identified the first frame in which participants completely left the force plate and the first frame in which any foot came back into contact with the force plate as per the instructions outlined by MyJump2 for the calculation of jump height (JH_MJ_).

Participants completed three experimental trials, each separated by 60 s of rest. Each jump was included in the analysis. The total sample for analysis was *n* = 89 jumps due to an error with force plate data capture for one trial.

### 2.3. Data and Statistical Analysis

Using raw unfiltered vertical force data as per the recommendations of Harry et al. [28], TIA was determined as the period between the participant leaving the force platform following the generation of vertical propulsion and initial contact with the force platform upon landing. A threshold of 10 N was used to objectively determine take-off and landing and the time difference between these phases was calculated to determine TIA [24,29,30]. Subsequently, JH_TIA_ was calculated using the equation [16]:

$${JH}_{TIA}=g\frac{{TIA}^{2}}{8}$$

*g = acceleration due to gravity* (9.81 m·s^−2^); *TIA = Time in the air*

As per the procedures outlined by [31], force-time data were mathematically integrated to determine velocity at each sampling point. TOV was determined as the final data point prior to take-off (i.e., the final point of the contact phase where data were <10 N) and JH_TOV_ calculated using the equation [16]:

$${JH}_{TOV}=\frac{{TOV}^{2}}{2g}$$

*g = acceleration due to gravity* (9.81 m·s^−2^); *TOV = Take-off Velocity*

In several cases, the data violated the normal distribution, assessed via Shapiro–Wilk (W > 0.93) and was confirmed following a visual inspection of *Q-Q* plots. However, when just the female data were considered, the data were approximately normally distributed. Friedman’s test was performed to evaluate differences between the methods used to determine jump height, where significant main effects were explored using Bonferroni corrected pairwise comparisons. A single-factor Analysis of Variance (ANOVA) was used to evaluate differences between the methods used to determine jump height when just the female data were examined. Mann–Whitney U tests to evaluate differences between males and females. Effects sizes (*r*) were calculated as per the equation below [32] and were interpreted as small (>0.1), medium (>0.3) and large (>0.5) [33].

$$r=\frac{Z}{\surd n}$$

Comparison of the performance of the MyJump2 to force-plate-derived measures of CMJ height were determined by calculating intraclass correlation coefficients (ICC), coefficient of variation (CV), standard error of measurement (SEM), and by conducting Bland–Altman analysis. ICC and 95% confidence limits were calculated using a single measure two-way mixed model for absolute agreement [34]. The ICC is a common statistical assessment used to determine reliability, defined as the extent to which a measurement can be replicated, with the ICC reflecting the degree of correlation and agreement between measurements [34]. ICCs were interpreted as poor (<0.5), moderate (<0.75), good (<0.9) or excellent (>0.9) [34]. For each individual, the between tool CV was determined as Mean/SD*100 and the Mean CV for each comparison reported. A CV < 5% was set as the criterion to declare measurement between tools as comparable [35]. Similarly, for each individual the between tool SEM was calculated as the SDdiff/√2, where SDdiff is the SD of the individual between tool differences [36]. The mean SEM for each comparison is reported. For Bland–Altman plots, mean bias between tools and 95% limits of agreement (LoA) were determined [37]. These analyses were repeated to assess between observer reliability to account for observer error influencing the performance of MyJump2, where identification of take-off and landing may differ between assessors.

Statistical significance was a priori set at an alpha level of *p* < 0.05. SEM and CVs were calculated in Microsoft Excel (v2016, Microsoft Corporation, Washington, DC, USA). ANOVA and ICC were conducted using SPSS V.26 for windows (SPSS Statistics for Windows, IBM Corp, Armonk, NY, USA). Bland–Altman and graphical representation of data were performed using GraphPad Prism (Version 8.3.1, San Diego, CA, USA).

## 3. Results

**Figure 1:**
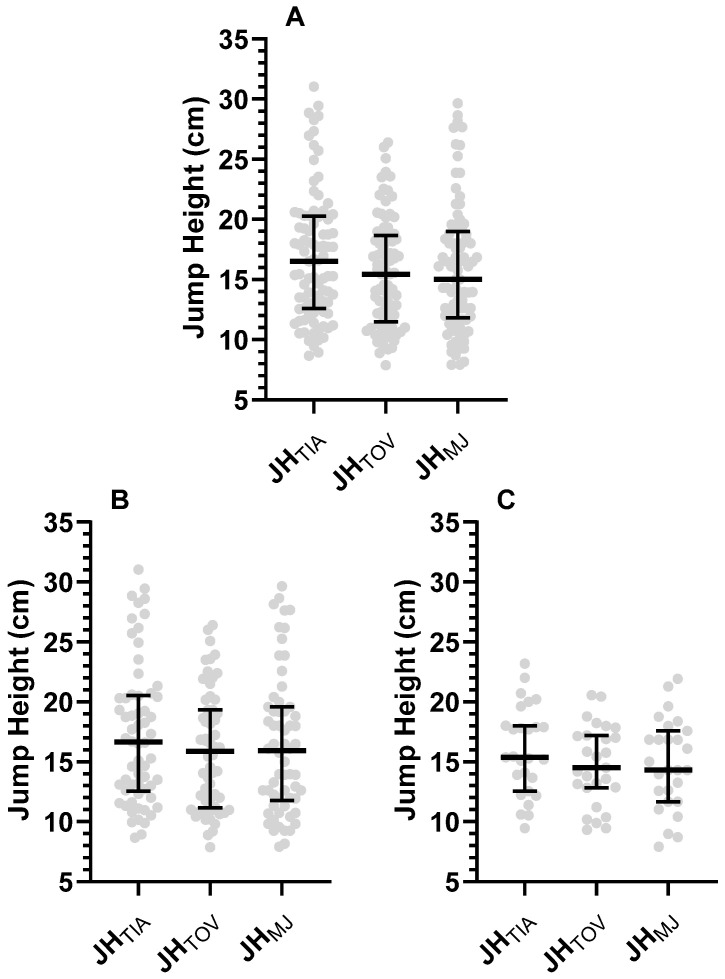
CMJ performance determined as JH_TIA_, JH_TOV_, JH_MJ_ [(**A**) = All participants; (**B**) = Males; (**C**) = Females; Data represented as Median ± Interquartile Range; grey circles represent individual data points; *n* = 89; JH_TIA_ = jump height calculated from the force plate from time in the air; JH_TOV_ = jump height calculated from the force plate from take-off velocity; JH_MJ_ = jump height calculated from MyJump2; All participants Median(IQR); JH_TIA_ 16.6 (20.7–12.2) cm, JH_TOV_ 15.5 (18.7–10.9) cm, JH_MJ_ 15.0 (19.2–12.6) cm; Males; JH_TIA_ 16.6 (20.7–12.7) cm, JH_TOV_ 16.2 (19.3–10.9) cm, JH_MJ_ 15.8 (19.2–12.0) cm; Females JH_TIA_ 15.3 (17.7–12.2) cm, JH_TOV_ 14.5 (17.2–13.2) cm, JH_MJ_ 14.3 (18.0–11.7) cm].

Kruskal–Wallis test revealed no difference in the measured jump height between the assessment tools (Figure 1A. *p* = 0.272) with a trivial effect size between JH_MJ_ and JH_TOV_ (*r* = 0.009), and small effect sizes between JH_TIA_ and both JH_MJ_ (*r* = 0.100) and JH_TOV_ (*r* = 0.106), where JH_TIA_ was greater. Irrespective of the assessment tool, jump height did not differ between males and females (Figure 1B,C. *p* > 0.381; *r* < 0.093). When stratified by sex, jump height did not differ between the assessment tools (Figure 1B,C. *p* > 0.39). For males, effect sizes between JH_MJ_ and both JH_TIA_ and JH_TOV_ were trivial (*r* = 0.094 and *r* = 0.013, respectively), and the effect size between JH_TOV_ and JH_TIA_ small (*r* = 0.107), where JH_TIA_ was greater. For females, the effect size between JH_MJ_ and JH_Tov_ was trivial (*r* = 0.003) and the effect size between JH_TIA_ and both JH_MJ_ and JH_TOV_ were small (*r* = 0.143 and *r* = 0.146, respectively), where JH_TIA_ was greater.

**Figure 2:**
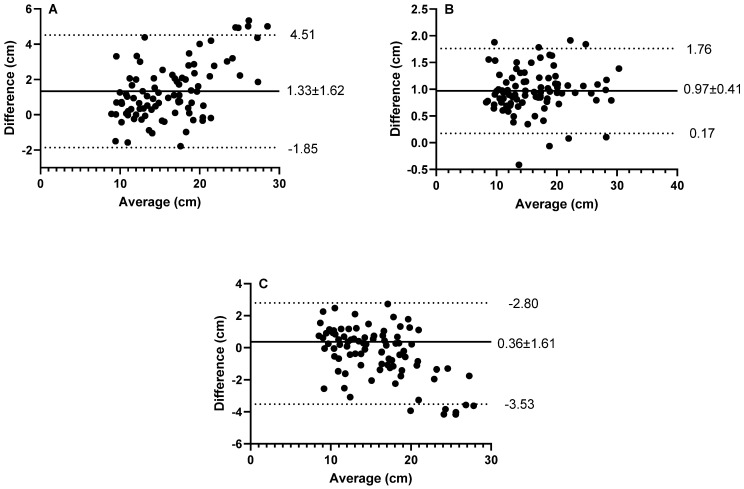
Bland–Altman Plots for JH_TIA_ vs. JH_TOV_ (**A**), JH_TIA_ vs. JH_MJ_ (**B**) and JH_TOV_ vs. JH_MJ_ (**C**). The centre lines represent the average difference between instruments and the upper and lower lines the upper and lower limits of agreement [*n* = 89].

There was excellent agreement between the JH_TIA_ and JH_TOV_ (Table 1. ICC = 0.955 (95% CI 801–0.982); SEM = 1.1 cm). However, the CV was 6.6% (Table 1) and Bland–Altman plots indicated that JH_TIA_ overestimated jump height compared to JH_TOV_ by 1.33 ± 1.62 cm (Figure 2A. LoA = −1.85–4.51 cm). When stratified by sex, there was excellent agreement between the JH_TIA_ and JH_TOV_ for both males (Table 1. ICC = 0.956 (95% CI 0.823–0.982); SEM = 1.3 cm) and females (Table 1. ICC = 0.945 (CI’s 0.583–0.984); SEM = 0.8 cm). The CV was 6.8% and 6.0% (Table 1.) and Bland–Altman plots indicated that JH_TIA_ over predicted JH_TOV_ by 1.39 ± 1.80 cm and 1.19 ± 1.16 cm for males and females, respectively (LoA = −2.13–4.91 cm for males and −1.08–3.45 cm for females).

There was also excellent agreement between the JH_TIA_ and JH_MJ_ (Table 1. ICC = 0.990; SEM = 0.3 cm), however, the ICC 95% CI ranged from poor to excellent (Table 1. ICC 95% CI 0.322–0.998). The CV was below the predetermined threshold to determine agreement (Table 1. CV = 4.7%) with Bland–Altman plots indicated that JH_TIA_ overestimated jump height compared to JH_MJ_ by 0.97 ± 0.41 cm (Figure 2B. LoA = 0.17–1.76 cm). When stratified by sex, there was excellent agreement between the JH_TIA_ and JH_TOV_ for both males (Table 1. ICC = 0.993 (95% CI 0.381–0.999); SEM = 0.3 cm) and females (Table 1. ICC = 0.972 (95% CI 0.078–0.955); SEM = 0.3 cm). The CV was 4.2% and 5.5% (Table 1) and Bland–Altman plots indicated that JH_TIA_ overestimated jump height compared to JH_TOV_ by 0.90 ± 0.40 cm for males and 1.11 ± 0.47 cm females, respectively (LoA = −0.20–1.61 cm for males and −0.19–2.04 cm for females).

The agreement between JH_TOV_ and JH_MJ_ was also excellent (ICC = 0.971 (95% CI = 0.956–0.981); SEM = 1.1 cm). The CV was slightly above the 5% threshold (Table 1. CV = 5.7%) and Bland–Altman plots indicated that JH_MJ_ overestimated jump height compared to JH_TOV_ by 0.36 ± 1.61 cm (Figure 2C. LoA = −3.52–2.80 cm). When stratified by sex, there was excellent agreement between the JH_TIA_ and JH_TOV_ for both males (Table 1. ICC = 0.972 (95% CI 0.952–0.983); SEM = 1.2 cm) and females (Table 1. ICC = 0.967 (95% CI 0.928–0.985); SEM = 0.6 cm). The CV was 6.1% and 4.7% (Table 1) and Bland–Altman plots indicated that JH_TIA_ overestimated jump height compared to JH_TOV_ by 0.49 ± 1.73 cm for males and 0.08 ± 1.29 cm females, respectively (LoA = −3.87–2.90 cm for males and −2.60–2.44 cm for females).

**Figure 3:**
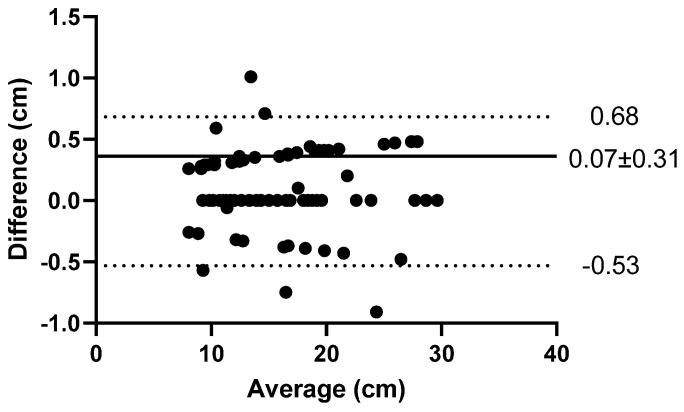
Bland–Altman Plot for JH_MJ_ determined by observer 1 and observer 2 [*n* = 89].

There was excellent agreement between observer 1 and observer 2 (ICC = 0.975 (95% CI 0.961–0.983); *p* < 0.001; SEM = 0.22 cm). The CV was low (1.0%) as was the mean bias (Figure 3. 0.07 ± 0.31 cm) and limits of agreement (Figure 3. −0.53–0.68 cm).

## 4. Discussion

The purpose of the present study was to provide novel insight with respect to the agreement between CMJ height measured from MyJump2 smartphone application and that derived from force platforms (determined by both TIA and TOV) in youth grassroots soccer players. Results infer agreement between jump height assessment methods; however, JH_TIA_ was higher than JH_TOV_ with a small effect size. The greatest CV (6.6%), systematic bias (1.33 ± 1.62 cm) and largest limits of agreement (−1.85–4.51 cm) were found between JH_TOV_ and JH_TIA_. Despite similar performance between JH_MJ_ and JH_TIA_, Bland–Altman analysis indicated a small systematic bias where JH_TIA_ overestimated jump height compared to JH_MJ_. This small systematic error resulted in JH_MJ_ performing better than JH_TIA_ when compared to the gold standard JH_TOV_. Jump height did not differ between males and females and the results demonstrate that the comparison between assessment tools was not sex specific. Whilst the results infer value in smartphone applications for measuring jump height in youth athletes, caution is needed when deriving jump height from TIA methods, as the seemingly small reported absolute systematic bias may represent large error when considered relative to the low jump heights (median 15.5 cm) achieved in this population.

The agreement between JH_TIA_ and JH_MJ_ in the present study is in keeping with previous work mainly in adult populations, which has assessed the validity of MyJump compared to other TIA-derived measures of jump height, including force platforms [17,18,19,20,21,22,23]. One key point of difference is the larger ICC 95% CI’s seen in the present study compared to previous work (0.322–0.998), which at the lower end has shown to be greater than 0.680 [26] but typically greater than 0.990 for CMJ assessments [17,18]. Despite this, JH_TIA_ compared JH_MJ_ demonstrated low CV and systematic bias and 95% LoA’s, adding to the body of the literature indicating that MyJump is valid when compared to TIA-derived jump height from a force platform.

However, the value of any instrument to accurately measure the desired outcome should be considered with respect to the gold standard, which in this case is jump height determined from TOV [16]. Despite agreement between JH_Tov_–JH_TIA,_ JH_Tov_ was lower than JH_TIA_ with a small effect size. Furthermore, the systematic bias and 95% LoA were higher than all other comparisons. Whilst a mean bias of 1.33 cm and LoA ranging between −1.85 and 4.51 cm would appear small, in the context of the youth population where the median JH_Tov_ was 15.5 cm, this may mean that in some cases, JH_TIA_ may substantially overestimate jump height. CMJ is a complex multi-joint movement requiring complex motor coordination [5], where accurate execution develops with age and maturation and subsequently is biomechanically variable [27,38]. Given accurate measurement of jump height from TIA requires almost perfect kinematics, where take-off and landing position are identical [16], this is likely the source of error between methods of assessment. More specifically, vertical jump landing position is typically lower than the take-off position due to preparatory hip, knee and ankle flexion to attenuate impact force on landing making it challenging to achieve the assumed parabolic trajectory of the CoM used in in the equation of motion associated with the TIA method [25].

The present work is the first to compare performance of the MyJump app to TOV-derived jump height in a youth population. Surprisingly, JH_MJ_ performed better than JH_TIA_, when compared to JH_TOV_. Previous work that has compared the performance of MyJump to force-plate-derived TOV measures of jump height in adults is sparse, and the results are equivocal [18,26], whilst the data in the present study would appear to agree with the findings of Carlos-Vivas et al. [18], who demonstrated excellent agreement despite a systematic bias where MyJump overestimated jump height derived from TIA. Interestingly, Armada-Cortés et al. [26] contradicted the findings of the present and previous work [18], showing poor agreement and a significant mean difference between measures. Armada-Cortés et al. [26] conducted validity analysis for combined SJ and CMJ. It may be that poor validity was influenced by bigger discrepancies in SJ performance, given that mean differences between instruments were demonstrated here and not between CMJ assessments.

The improved performance of the JH_MJ_ compared to JH_TIA_ is likely attributable to differences in identifying take-off and landing. Whilst this is carried out objectively for force platform analysis using a threshold force value that is believed to be greater than the noise in the force trace to identify the period when the performer is off the ground, there is both an objective and subjective nature to the evaluation of TIA using video analysis. Whilst clear objective criteria are defined (as per that outlined in the method), there is a subjective nature to defining the frame which the performer leaves and returns to the force plate which is almost certainly the nature of the small between-rater differences outlined here and in previous work [17]. Furthermore, this is likely compounded by a higher sampling rate in the force plate (1000 Hz) compared to the high-speed camera used to capture image data to be processed with MyJump. As previously outlined, the lower sampling frequency used for video capture likely results in a small magnitude of error in identifying the exact time in which the performer is in contact with the force platform [17].

When stratified by sex, there was no difference in jump height between males and females, and the agreement between the tools did not appear to be sex-specific. Previous work in children of a similar age to that was used in the present study indicates that the development of qualitative elements of technique in fundamental movement skills can be sex-specific, with enhanced development of locomotor skills in males and object control skills in females [39,40]. However, in many cases, these differences are determined when several locomotor and object control skills are considered collectively, and sex-specific differences in motor development are less apparent when examined at an individual skill component level [39]. Given that the accuracy of TIA-derived jump height relies on the precise execution of in-flight technique, a lack of superior sex-specific motor development in this skill may explain the comparable agreement between tools for males and females seen in the present study. Previous work indicates that the reliability of CMJ performance in both males and females, an indicator of motor competence, improves after the age of 10 years [41], which may result in an improved agreement between tools. Despite this, sex-specific differences in the agreement between tools may be apparent at different stages of maturity, given that superior development of jump performance in males has been shown to occur beyond the age of 10 years [41]. If the same technique is executed, the absolute error of jump height derived from TIA will be increased if greater jump performance is achieved, and thus agreement between tools and methods of calculation may be subject to sex-specific differences, particularly if jump technique is not fully developed.

### Limitations and Future Directions

Despite the present study offering important insight into the accuracy of the assessments of vertical jump performance in youth, it is not without limitations. The results obtained from the ICCs may need to be interpreted with caution, given the small deviations from normal distribution that were reported for some of the data. However, given that the study uses several statistical approaches to determine the agreement and magnitude of error between assessment methods, confidence should be taken in the conclusions. Moreover, the trends seen in the ICCs reflect those demonstrated in the other statistical analysis and given that ICCs are commonplace in previous work evaluating the agreement between MyJump and force-platform-derived jump height, their inclusion is important for comparison to previous work.

Participants in the present study wore their own athletic training shoes, and thus, footwear was not standardised. Although there is evidence in adult populations to suggest that footwear may influence jumping performance [42], this will have little impact on the ability of the tools used in the present study to measure jump height. Finally, within-session reliability was not determined given that, as per our previous work [29], vertical jump performance may be impacted by poorer reliability in youth compared to adult populations which would impact within-session reliability assessment of the same tool given that this analysis relies on the comparison of different jumps from the same participant.

Whilst the systematic error in MyJump may be beneficial in the case of comparison to jumping height derived from TOV, it may only be specific to 240 Hz sampling frequency and in the current population, where jump height is low (compared to adult populations used in the previous literature), and the movement pattern is less well established. Furthermore, conclusions of the present study are limited to CMJ assessments, and there is a future need to evaluate the performance of TIA measures of jump height, and more specifically MyJump, for SJ and drop jump performance, where the performance of TIA equations may differ inferred by previous work [26]. Additionally, the present findings and previous work in the area are limited to the evaluation of the performance of MyJump for the assessment of bilateral vertical jumps. Given most of the maximal activities during soccer match play (i.e., sprinting, cutting, jumping) rely on unilateral force ground contact, evaluating unilateral vertical jump performance is important to lower-limb power profiling of athletes and has several further important applications such as the identification of bilateral strength asymmetries [43,44]. Compared to bilateral vertical jumps, unilateral jump performance is both biomechanically distinct and the movement pattern less repeatable in youth [29]; thus, investigation of the accuracy of MyJump for quantifying unilateral jump performance is warranted.

## 5. Conclusions

Given the low jump heights achieved in youth and the potential lack of movement competency, TIA methods to determine CMJ height should be used with caution, despite intraclass correlation coefficients identifying excellent agreement between methods. Given the importance of vertical jump assessment as a benchmarking tool, assessment of neuromuscular function, and indicator of health status, force-plate-derived TOV should be used to guarantee accuracy in the calculation of jump height. Where this is not possible, MyJump2 may be a suitable alternative, but participant movement competency should first be established, and a careful evaluation of take-off and landing mechanics is needed to help reduce the systematic bias of this instrument.

## Figures and Tables

**Table 1:** CV, SEM and ICC of CMJ performance determined as JH_TIA_, JH_TOV_, JH_MJ_.

		CV%	SEM (cm)	ICC (95%CI)	ICC *p*
All	JH_TIA_ vs. JH_TOV_	6.6	1.1	0.955 (0.801–0.982)	<0.001
	JH_TIA_ vs. JH_MJ_	4.7	0.3	0.990 (0.322–0.998)	<0.001
	JH_TOV_ vs. JH_MJ_	5.7	1.1	0.971 (0.956–0.981)	<0.001
Males	JH_TIA_ vs. JH_TOV_	6.8	1.3	0.956 (0.823–0.982)	<0.001
	JH_TIA_ vs. JH_MJ_	4.2	0.3	0.993 (0.381–0.999)	<0.001
	JH_TOV_ vs. JH_MJ_	6.1	1.2	0.972 (0.952–0.983)	<0.001
Females	JH_TIA_ vs. JH_TOV_	6.0	0.8	0.945 (0.583–0.984)	<0.001
	JH_TIA_ vs. JH_MJ_	5.5	0.3	0.972 (0.078–0.955)	<0.001
	JH_TOV_ vs. JH_MJ_	4.7	0.9	0.967 (0.928–0.985)	<0.001

## Data Availability

Data is available upon request.
